# IMP/GTP balance modulates cytoophidium assembly and IMPDH activity

**DOI:** 10.1186/s13008-018-0038-0

**Published:** 2018-06-15

**Authors:** Gerson Dierley Keppeke, Chia Chun Chang, Min Peng, Li-Yu Chen, Wei-Cheng Lin, Li-Mei Pai, Luis Eduardo Coelho Andrade, Li-Ying Sung, Ji-Long Liu

**Affiliations:** 1 Department of Physiology, Anatomy and Genetics, University of Oxford, Oxford, OX1 3PT UK; 2 Institute of Biotechnology, National Taiwan University, Taipei, 106 Taiwan, ROC; 3 Molecular Medicine Research Center, College of Medicine, Chang Gung University, Tao-Yuan, 333 Taiwan, ROC; 4 Graduate Institute of Biomedical Science, College of Medicine, Chang Gung University, Tao-Yuan, 333 Taiwan, ROC; 5 Department of Biochemistry, College of Medicine, Chang Gung University, Tao-Yuan, 333 Taiwan, ROC; 6 Rheumatology Division, Escola Paulista de Medicina, Universidade Federal de Sao Paulo, Sao Paulo, SP 04023-062 Brazil; 7 Agricultural Biotechnology Research Center, Academia Sinica, Taipei, 115 Taiwan, ROC; 8 School of Life Science and Technology, ShanghaiTech University, Shanghai, 201210 China

**Keywords:** Cytoophidium, IMPDH enzyme, GTP biosynthesis, IMP, Cell proliferation

## Abstract

**Background:**

Inosine monophosphate dehydrogenase (IMPDH), the rate-limiting enzyme in de novo GTP biosynthesis, plays an important role in cell metabolism and proliferation. It has been demonstrated that IMPDH can aggregate into a macrostructure, termed the cytoophidium, in mammalian cells under a variety of conditions. However, the regulation and function of the cytoophidium are still elusive.

**Results:**

In this study, we report that spontaneous filamentation of IMPDH is correlated with rapid cell proliferation. Intracellular IMP accumulation promoted cytoophidium assembly, whereas elevated GTP level triggered disassociation of aggregates. By using IMPDH2 CBS domain mutant cell models, which are unable to form the cytoophidium, we have determined that the cytoophidium is of the utmost importance for maintaining the GTP pool and normal cell proliferation in the condition that higher IMPDH activity is required.

**Conclusions:**

Together, our results suggest a novel mechanism whereby cytoophidium assembly upregulates IMPDH activity and mediates guanine nucleotide homeostasis.

**Electronic supplementary material:**

The online version of this article (10.1186/s13008-018-0038-0) contains supplementary material, which is available to authorized users.

## Background

Purine nucleotides are essential molecules for a variety of cell functions. They not only serve as the building blocks of DNA and RNA, but also participate in cell metabolism as cofactors or energy donors, and are involved in cell signal transduction and cytoskeleton organisation. Imbalance of nucleotide pools may lead to cell cycle arrest, mistakes in nucleic acid synthesis or even cell death. Thus, the precise regulation of nucleotide biosynthesis is crucial for regular cell metabolism [1].

Purine nucleotides can be synthesized by a salvage pathway or a de novo synthetic pathway. By the former purine free bases will be recycled by hypoxanthine/guanine phosphoribosyl transferase (HGPRT), adenine phosphoribosyl transferase (APRT) or adenosine kinase (ADK), whilst by the latter the purine ring is assembled in serial steps from precursors of carbohydrate and amino acid metabolism.

Inosine monophosphate (IMP) is a common precursor in adenine and guanine nucleotide de novo synthetic pathways. IMP dehydrogenase (IMPDH) catalyses the NAD-dependent conversion of IMP to xanthine monophosphate (XMP), the rate-limiting step of guanine nucleotide synthesis. Because of its pivotal role in nucleotide production, IMPDH inhibitors have been applied in therapies for auto-immune diseases, infectious diseases and cancers. For example, mycophenolic acid (MPA), a metabolite of mycophenolate sodium or mycophenolate mofetil, works as an immunosuppressant and is widely used to prevent rejection upon organ transplantation and in the treatment of some forms of systemic lupus erythematosus (SLE) and vasculitis [2–4]. In addition, ribavirin is used in combination with interferon-α for the treatment of chronic hepatitis C virus infection (HCV), and tiazofurin was once an orphan drug for chronic myelogenous leukemia [5–8].

In mammals, two IMPDH isoforms, IMPDH1 and IMPDH2, are encoded by individual genes. They share 84% identical amino acid sequences and indistinguishable kinetic properties but differ in expression patterns [2, 9–11]. IMPDH2 is the predominant isoform in most tissues, whilst IMPDH1 is generally expressed at a low level except in certain tissues including retina, spleen and in resting peripheral blood mononuclear cells [12–14]. Although the tetramer is a stable state for IMPDH, the formation of IMPDH octamers and even higher order polymers has been shown in recent studies [15–18]. IMPDH contains a catalytic domain and a subdomain consisting of two repeated cystathionine β-synthase (CBS) domains. Despite its dispensable role for catalytic activity in vitro, the CBS domain has been demonstrated as responsible for mediating the allosteric inhibition and polymerisation of IMPDHs [12, 17–20].

The cytoophidium (plural: cytoophidia) is an organelle-like macrostructure comprised of metabolic enzymes [21–23]. The term cytoophidia comes from the Greek for “cellular snakes”, although these structures are also known as Rods and Rings (RR) from its shape [24–26]. In mammalian cells, IMPDH and cytidine triphosphate synthase (CTPS) are two identified components of the cytoophidium [15, 24, 27, 28]. By electron microscopic analysis, it has been shown that the cytoophidium is composed of a huge bundle of protein polymer fibres [17, 29, 30]. Although IMPDH and CTPS are able to form the cytoophidium independently, their colocalisation is frequently observed [24, 31, 32].

Despite the inhibitory effect of CTPS activity from polymerization has been reported in bacterial models, a more recent study shows a converse scenario on purified human CTPS1 [33, 34]. As the mutant CTPS without the ability to polymerize displayed significantly lower catalytic activity in vitro, the function of polymerization for upregulation of CTPS activity has been demonstrated [33]. In contrast, an in vitro study suggests that polymerization of human IMPDH is independent of the regulation of its catalytic activity [35]. However, results from purified proteins could not fully represent the whole picture of the cytoophidium, which is a much larger and probably much more complicated structure than protein polymers. Further investigation is required to learn the role of the cytoophidium in cell metabolism.

In this study, our focus will be on the IMPDH-based cytoophidium. In all mammalian cells tested so far, IMPDH cytoophidia could be induced by inhibitors that impede GTP biosynthesis, and disassembled by guanosine or GTP supplementation [15, 20, 24]. However, the cytoophidium is also found in multiple cell types under drug-free culture conditions [31, 36]. For instance, the natural presence of IMPDH cytoophidia was detected in about 95% of mouse embryonic stem cells (ESCs) [24]. Moreover, IMPDH spontaneously forms cytoophidia in mouse pancreatic islet cells in response to nutrient uptake [31]. Since GTP participates in the regulatory pathway of insulin secretion, the physiological correlativity of the IMPDH cytoophidium has been proposed [31, 37]. Observations to date suggest that IMPDH cytoophidium assembly is a natural biological phenomenon involving purine metabolism, although its regulation and function remain largely unclear.

Herein, we aim to explore the regulation and the fundamental function of the IMPDH cytoophidium. We firstly show that the spontaneous formation of the IMPDH cytoophidium in mouse induced pluripotent stem cells (iPSCs) is correlated with rapid cell proliferation. Secondly, we determine that IMPDH cytoophidium assembly is promoted by its substrate IMP. Finally, by applying the CRISPR/Cas9 genome editing system, we established IMPDH2 CBS domain mutant HeLa cell lines with no cytoophidium-forming capability. With these distinctive model cells, we discovered that formation of the cytoophidium, by IMPDH1 and IMPDH2, is required for producing sufficient amounts of guanine nucleotides to maintain normal cell proliferation when the intracellular IMPDH level is not adequate. Collectively, we propose that formation of the cytoophidium is a novel mechanism to boost IMPDH activity and mediate nucleotide homeostasis.

## Methods

### Cell culture and transfection

HEK 293T cells and HeLa cells (obtained from Culture Collections, Public Health England #93021013) were cultured in DMEM with high glucose (Thermo Fisher Scientific) and supplemented with 10% FBS (Thermo Fisher Scientific), 2 mM l-Glutamine and 1% Gibco^®^ Antibiotic–Antimycotic (Thermo Fisher Scientific). Mouse iPSCs, which were kindly provided by Prof. Paul Fairchild (from Sir Willian Dunn School of Pathology, University of Oxford), were cultured without feeders in a surface coated with 0.1% porcine skin gelatin with a homemade medium: DMEM (Sigma #D6546), 15% FBS batch tested (Gibco #16000-036), 2 mM l-Glutamine, 1% Penicillin/Streptomycin (Gibco), 1% Non-Essential amino acids (Gibco), 0.1% of β-Mercaptoethanol stock solution (obtained by dilution of 70 µl 100% in 20 ml PBS), 1000 units/ml of mLIF (Millipore #ESG1107). Culture medium was replaced every 2 days for mouse iPSCs and pluripotency was monitored by characteristic colony formation. All cells were cultured in a 37 °C humid incubator with 5% CO_2_. Thymidine (Sigma #T1895), guanosine (Sigma #G6264), allopurinol (MP Biomedicals #0219015001) and MPA (Alfa Aesar #J61905) were dissolved in DMSO (Sigma-Aldrich). DON (Sigma #D2141), ribavirin (Abcam #ab120660), dCTP (Invitrogen #10217016) and GTP (Sigma #G8877) were dissolved in water. All drugs were applied in the concentrations as indicated. For cell transfection with constructs, lipofectamine 3000 reagent (Thermo Fisher Scientific) was used for HEK 293T cells and effectene transfection reagent (QIAGEN) was used for HeLa cells. All transfection procedures were according to instructions provided by manufacturers.

### Establishment and characterisation of mutant cell colonies with CRISPR/Cas9

The plasmid pU6-(BbsI)_CBh-Cas9-T2A-mCherry was a gift from Prof. Ralf Kühn (Addgene plasmid # 64324) [38], was used for generating mutant HeLa cell lines. In brief, the plasmid was digested with restriction enzyme Bpil (Thermo Fisher Scientific) and purified. Subsequently, sgRNA oligonucleotide was inserted into the opened plasmid with NEBuilder HiFi DNA Assembly Master Mix (New England Biolabs). Sequences of sgRNAs for IMPDH1 and IMPDH2 are shown in Additional file 1: Table 1. Two days after transfection, HeLa cells expressing mCherry were sorted and single cells were seeded into 96-well plate. About 2 weeks later, the DNA targeted regions as described in Results for the interested colonies were PCR amplified with primers shown in Additional file 1: Table 1, DNA fragments were cloned into carrier vectors with Gibson Assembly Master Mix (New England Biolabs) and used for DH5α bacterial transformation. Random colonies were picked and the vector extracted was sequenced for characterisation of individual alleles.

### Constructs

For myc-IMPDH1 and myc-IMPDH2 overexpression, the IMPDH1 and IMPDH2 coding sequences were amplified with primers shown in Additional file 1: Table 1 and inserted into pTRE2hyg plasmid (Clontech). For GMPR overexpression, the coding sequence (NCBI Reference Sequence: NM_006877.3) was amplified from HeLa total cDNA (See Real-time q-PCR methods) with primers shown in Additional file 1: Table 1 and inserted into linearized pCMV3-N-OFPSpark plasmid (Sino Biological), together with a DNA fragment containing the P2A sequence, with Gibson Assembly Master Mix (New England Biolabs). Mutant plasmids were generated with QuikChange II Site-Directed Mutagenesis Kit (Agilent) and protocols provided by the manufacturer.

For the concomitant overexpression of fluorescent-tagged IMPDH1 and IMPDH2, plasmids used were pEZ-M32-eCFP-N-IMPDH1 (GeneCopeia #EX-Z3998-M32) and pCMV3-OFPSpark-N-IMPDH2 (Sino Biological #HG14878-ANR).

### IMPDH2 knockdown

For the knockdown of IMPDH2 mRNA, a pool of four predesigned siGENOME SMARTpool siRNA IMPDH2 was used in the indicated concentrations (GE Dharmacon #M-004331-02-0010). Sequences are presented in Additional file 1: Table 1. As control, siGENOME Non-Targeting siRNA pool was transfected in the same concentrations as the target (GE Dharmacon #D-001206-13-05). For the transfection of siRNAs, Lipofectamine RNAiMAX Reagent (Thermo Fisher Scientific) was used following instructions provided by the manufacturer. siRNA transfection efficiency was monitored with siGLO green transfection indicator (GE Dharmacon) as shown in Additional file 1: Fig. 3d.

### EdU labelling

For EdU incorporation, 15–20 min before fixation cells were incubated with 20 µM of EdU. After 4% PFA fixation, a Click-iT^®^ azide-based reaction was performed to bind Alexa Fluor 647 molecule to the EdU incorporated to newly synthesized DNA. All procedures followed manufacturer protocol (Thermo Fisher Scientific).

### Nucleotide analysis by UPLC

Quantification of intracellular nucleotides was performed as described previously [31, 39]. In brief, 7 × 10^6^ cells were lysed in 80% methanol. After centrifugation at 13,000×*g* for 10 min, the supernatants were collected and dried. Pellets were resuspended in water and analyzed using Acquity Ultra Performance Liquid Chromatography (UPLC, Waters) interfaced with a PDA photodiode array (Waters).

### IMPDH enzyme activity measurement

IMPDH enzyme activity was measured in total cell extract for the given cell lines with an assay that is based on the reduction of INT in a NADH-coupled reaction to INT-formazan which exhibits an absorption maximum at 492 nm and allows for sensitive measurement of IMPDH activity in a plate reader. The assay was performed following the manufacturer recommendations (BMR Service #E-119; School of Medicine and Biomedical Sciences, State University of New York at Buffalo).

### Immunofluorescence

Immunofluorescence was performed as previously described [32]. Primary antibodies used: Rabbit Polyclonal anti-IMPDH2 antibody (ProteinTech, 12948-1-AP); Mouse monoclonal anti-IMPDH1 antibody (Abcam, ab55297); Mouse monoclonal anti-c-Myc antibody (Santa Cruz Biotech, sc-40). Secondary antibodies used: DyLight 488-Conjugated or Cy™3-Conjugated or DyLight 649-Conjugated Donkey Polyclonal anti-Mouse IgG (Jackson ImmunoResearch #715-165-151; #715-485-151; #715-495-151). Cy™3-Conjugated Donkey Polyclonal anti-Rabbit IgG (Jackson ImmunoResearch #711-165-152); Alexa Fluor^®^ 488-Conjugated or Alexa Fluor^®^ 647-Conjugated Donkey Polyclonal anti-Rabbit IgG (Invitrogen Mol Probes #A-21206; #A-31573). After the immunofluorescence probing, cells were analysed and images captured with a Leica TCS SP5 Confocal microscope.

### Immunoblotting

After the indicated treatment, cells were suspended with trypsin, washed once with PBS and lysed with RIPA buffer added of Protease inhibitor Cocktail (Thermo Fisher Scientific). Further homogenization was obtained by submitting the samples to five medium intensity cycles of sonication with 30 s each cycle. Cell extract was immediately stored in − 80 °C. Protein quantitation was obtained with BCA Protein Assay Kit (Thermo Fisher Scientific). Samples were submitted to denaturation in 95 °C for 10 min in the presence of Laemmli SDS sample buffer (Alfa Aesar). About 10 µg of protein was loaded in each well of 15/wells NuPAGE™ Bis–Tris gels, run with XCell SureLock™ Mini-Cell Electrophoresis System and transfer to nitrocellulose membrane with XCell II™ Blot Module (Thermo Fisher Scientific). After 2 h blocking with TBS + 5% milk, primary antibodies diluted in TBS + 5% milk were incubated overnight for 1 or 2 nights at 4 °C. After three times washing of the membrane with TBS, secondary antibodies were incubated overnight for 1 night in the same conditions. Antibody labelling was revealed with SuperSignal™ West Pico Chemiluminescent Substrate (Thermo Fisher Scientific) and visualized in a G:BOX Chemi XT4 machine (Syngene).

Primary antibodies used: Rabbit Polyclonal anti-IMPDH2 (ProteinTech #12948-1-AP); Mouse monoclonal anti-IMPDH1 (Abcam #ab55297); HRP-Conjugated Mouse monoclonal anti-ACTB or anti-GAPDH (ProteinTech #HRP-60008; #HRP-60004). Secondary antibodies used: HRP-Conjugated Donkey Polyclonal anti-Mouse IgG or anti-Rabbit IgG (Jackson ImmunoResearch #715-035-150; #711-035-152).

### Real-time PCR

After the appropriate transfection or treatment as indicated in Results, the cells were suspended with trypsin, centrifuged, and the RNA was extracted using miRNeasy Mini Kit (Qiagen #217004;) according to the manufacturer’s protocol. The amount of RNA obtained was quantitated by NanoDrop 2000c. RNA samples were immediately stored in a − 80° freezer. Reverse Transcriptase (RT) conversion of RNA to cDNA was made with Maxima First Strand cDNA Synthesis Kit with dsDNase (Thermo Fisher Scientific #K1671) according to the manufacturer’s protocol. The cDNA was stored in a − 20° freezer until the qPCR reaction. Quantitative PCR (qPCR) reaction was performed using 7500 Fast Real Time PCR System (Applied Biosystems), with SYBR Green ReadyMix (Sigma #S4438) as amplification indicator according to manufacturer protocol. Standard 60° Tm annealing temperature and 40 amplification cycles was used for all primer pairs. The quality of reaction was evaluated by Melt curve. Each sample was run in duplicate or triplicate. Target Ct genes were analysed by comparison with housekeeping references through ΔΔCt method. GAPDH was used as housekeeping reference. Primers used are presented in Additional file 1: Table 1.

### Image analysis and statistical comparisons

Images captured by the microscope were analysed using ImageJ 1.51 software. Proportion of cells presenting the given characteristic, such as cytoophidium presence or EdU labelling, was obtained by quantification of at least two randomly captured images (> 200 cells) in each of at least two independent experiments, unless stated otherwise. All western blot experiments were repeated at least twice with independent samples, and the intensity of the band was quantified using the Surface Plot tool in ImageJ software, normalized for the housekeeping reference. Average size of cytoophidium was estimated with the Analyse Particles tool in ImageJ, with a threshold value arbitrary settle to 50. The classification as high or low fluorescence intensity for cells expressing OFP (for example OFP-P2A-GMPR_wt) was obtained using ImageJ with a threshold settle at 50.

The data is presented as Mean plus error bars indicating Standard Deviation (SD) or Standard Error of the Mean (SEM) as described in figure legends. For the statistical comparison of quantitative and semi-quantitative parameters of different time-points of the same treatment, paired Student’s *t* test was used. For the comparison of different groups and/or different treatments, unpaired Student’s *t*-test was used. *P *≤ 0.05 was considered statistically significant. All analyses were done with Microsoft Office Excel^®^ or GraphPad Prism 5.0 software.

## Results

### IMPDH-based cytoophidium assembly in iPSCs responds to GTP levels and proliferation arrest

IMPDH cytoophidia can be induced both in vivo and in vitro under various conditions that interfere with de novo GTP synthesis, including treatment with IMPDH inhibitors, such as azathioprine, MPA and ribavirin, and others, including methotrexate, acyclovir, decoyinine, deazauridine (DAU) and 6-diazo-5-oxo-L-norleucine (DON) [15, 20, 24, 31, 40]. However, IMPDH cytoophidia were also observed in certain cell types without such drug stimulation [24, 31, 36]. To further explore the correlation between cytoophidium formation, purine metabolism and cell proliferation, we attempted to analyse the cytoophidium in a cell model with a high amount of IMPDH cytoophidia under normal culture conditions.

**Fig. 1:**
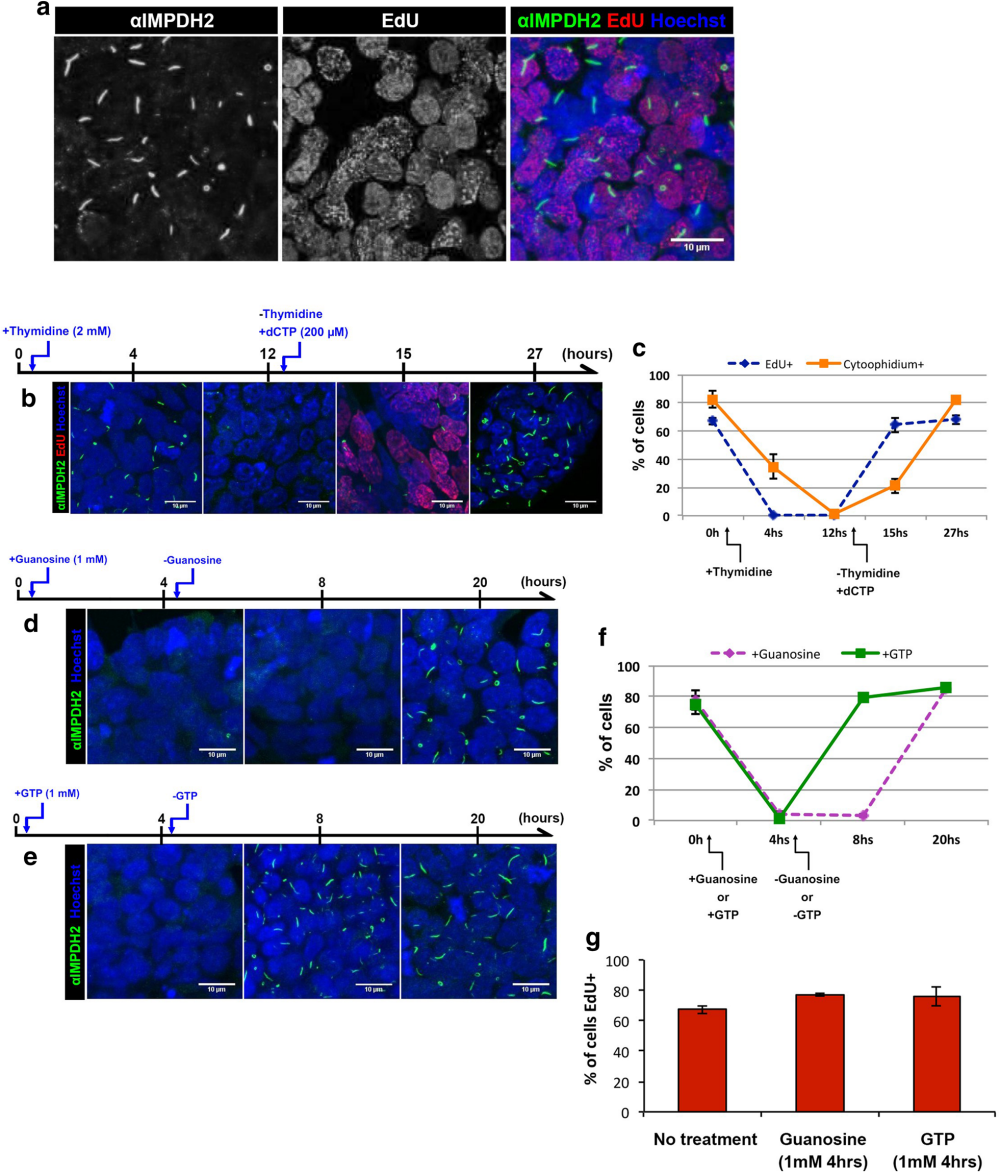
IMPDH-based cytoophidia in iPSCs respond to GTP levels and proliferation arrest. **a** iPSCs were labelled with anti-IMPDH2 antibody and EdU. **b** Cytoophidia disassembled completely in 12 h of 2 mM thymidine treatment. Once thymidine was removed and dCTP was added, cytoophidia reassembled in 12 h. **c** Quantitative results of conditions in **b**. **d** Cytoophidia disassembled when cells were treated with 1 mM guanosine for 4 h. After removal of guanosine, cytoophidia reassembled in 12 h. **e** With 1 mM GTP supplementation, cytoophidia disassembled in 4 h and reassembled in 4 h after removal of GTP. **f** Quantitative results of conditions in **d** and **e** indicating the proportion of cells with cytoophidium. **g** Proportion of cells labelled by EdU after 4 h of guanosine or GTP treatment. Mean (± SEM) is presented in **c**, **f** and **g** from at least 200 cells counted for each time point of the treatments in at least two independent experiments

A previous report shows that mouse embryonic stem cells (ESCs) present IMPDH-based cytoophidia [24]. In this study, we firstly labelled IMPDH in mouse iPSCs and observed IMPDH cytoophidia in 82 ± 5.9% of the cells under normal culture condition (Fig. 1a, c). This shows that IMPDH cytoophidium assembly is a natural phenomenon in iPSCs. Therefore, we aimed to investigate the correlation between IMPDH cytoophidium regulation and one of the characteristics of iPSCs, the rapid proliferation. Firstly, we treated cells with 2 mM of thymidine to arrest the cell cycle in G1/S phase and labelled proliferating cells with ethynyl deoxyuridine (EdU). Although the proportion of EdU positive cells was dramatically reduced from 67 ± 2.4 to 0% after 4 h of thymidine treatment, IMPDH cytoophidia were still present in 35 ± 8.5% of cells and were only completely disassembled after prolonged treatment for 12 h (Fig. 1b, c). Subsequently, we removed the thymidine and added dCTP (200 µM) in culture medium to restore cell proliferation. Three hours later, the percentage of EdU positive cells rose to 64 ± 5.2%, while only 21 ± 5.0% of cells exhibited cytoophidia. Until 15 h after cell cycle was resumed, the cytoophidia had reassembled in 82.5 ± 2.7% of cells (Fig. 1b, c). Cell cycle arrest may result in a reduction of nucleotide consumption thereby increase the intracellular level of GTP, which may induce IMPDH cytoophidia disassembly.

**Fig. 2:**
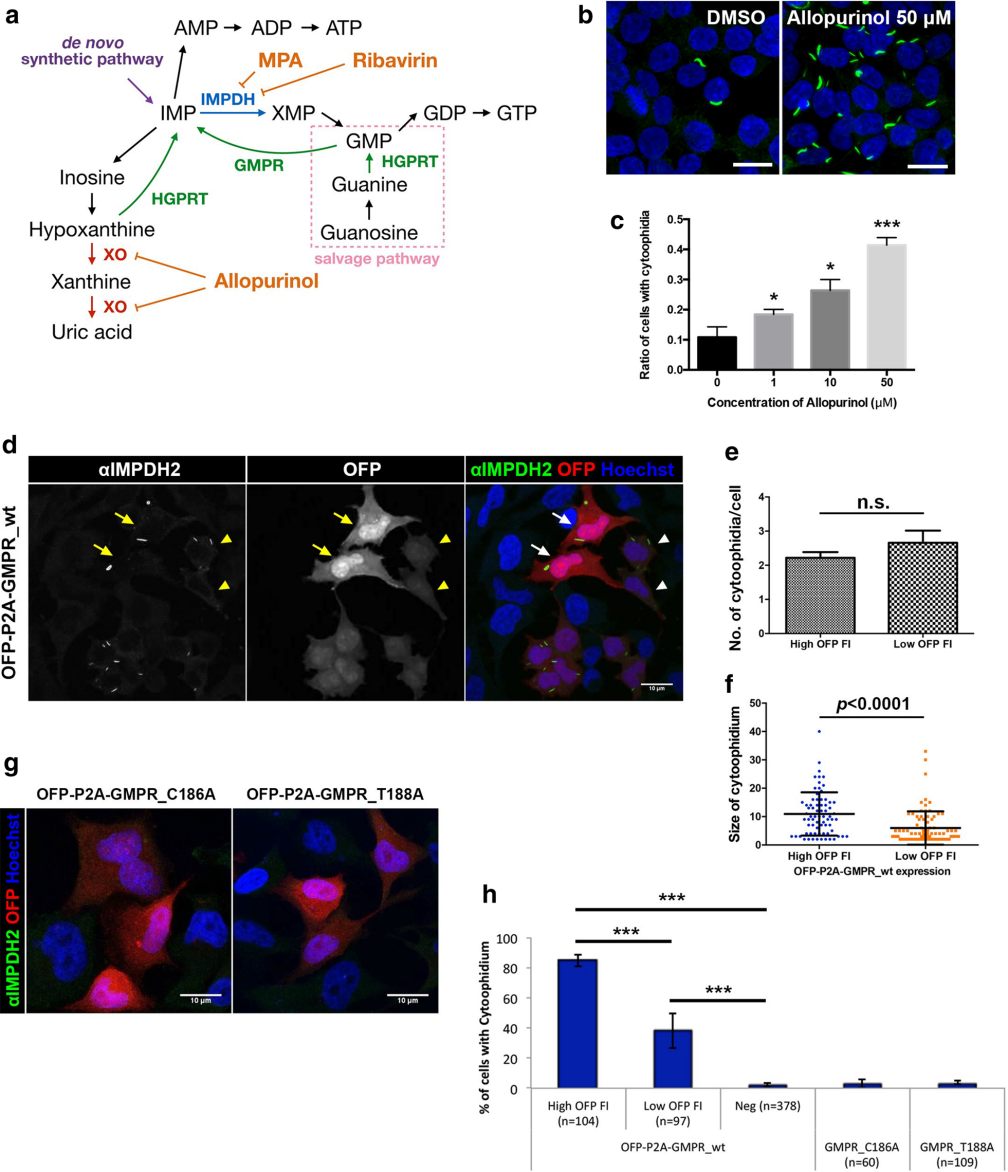
IMP promotes IMPDH cytoophidium formation. **a** A simplified diagram showing IMP-related metabolism. **b** Immunofluorescence for IMPDH2 in HEK 293T cells treated with DMSO or allopurinol (50 μM) for 1 h. **c** Mean (± SEM) percentages of HEK 293T cells with IMPDH cytoophidia after various doses of allopurinol treatment for 1 h. (Student’s *t*-test, *P < 0.05, ***P < 0.001). **d** Immunofluorescence of IMPDH2 antibody labelled IMPDH cytoophidia in OFP-P2A-GMPR-expressing HeLa cells. Cells with high fluorescence intensity indicated by arrows and cells with low intensity are indicated by arrowheads. **e** Average (± SEM) number of cytoophidia per cell in cells with high *versus* low OFP fluorescence intensity expressing OFP-P2A-GMPR_wt. **f** Average (± SD) size of cytoophidium in cells with high *versus* low OFP fluorescence intensity. **g** Immunofluorescence of HeLa cells expressing catalytic dead OFP-P2A-GMPR constructs. **h** Mean (± SD) of HeLa cells with IMPDH cytoophidia after transfection with different GMPR plasmids (Student’s *t*-test, ***P < 0.001). Nuclei were labelled with DAPI (blue) in all images and scale bar = 20 μm in **b** and 10 μm in **d** and **g**

In order to test whether the cytoophidia in iPSCs respond to an increase in GTP level, cells were treated with 1 mM guanosine. Having entered the cell, the guanosine would be converted into guanine by purine nucleoside phosphorylase (PNP), then into GMP by hypoxanthine guanine phosphoribosyl transferase (HGPRT) via the salvage pathway (Fig. 2a). After 4 h of guanosine treatment, cytoophidia were present in < 5% of cells (Fig. 1d, f). A similar result was observed in iPSCs treated with 1 mM GTP for 4 h (Fig. 1e, f), although treatment with dCTP for 4 h did not affect the cytoophidium in iPSCs (Additional file 1: Fig. 1). Interestingly, IMPDH cytoophidia reassembled within 12 and 4 h of removal of guanosine and GTP treatments, respectively (Fig. 1e, f). Meanwhile, the proliferation rate of iPSCs was not affected by guanosine or GTP treatment (Fig. 1g). These data indicate that the IMPDH cytoophidium is highly dynamic and associated with intracellular GTP level, as it disassembles when the GTP level is increased by an external source and reforms when GTP is back to the normal level within hours of the removal of additional guanosine or GTP. Our findings suggest the reason why IMPDH forms cytoophidia in iPSCs may be related to a high consumption of guanine nucleotides for supporting the rapid proliferation of these cells.

### IMP promotes IMPDH filamentation

We find that the high consumption of guanine nucleotides may be related with the presence of cytoophidium in iPSCs, evidenced by the rapid proliferation of these cells. We then suspect that, to support the de novo synthesis of guanine nucleotides, the substrate IMP supply would also be upregulated. This prompts us to examine whether IMP accumulation could promote IMPDH cytoophidium formation. Our previous study shows that the treatment with DAU leads to the temporal presence of IMPDH cytoophidia in HEK 293T cells with an elevated GTP level [31]. We suspected that is a consequence of IMP accumulation due to the inhibition of pyrimidine synthesis by DAU.

In mammalian cells, apart from the de novo synthetic pathway, IMP could also be converted from hypoxanthine and GMP by HGPRT and GMPR, respectively (Fig. 2a). First, we treated HEK 293T cells with allopurinol, which impedes hypoxanthine degradation by inhibiting xanthine oxidase, thereby enhances reutilization of hypoxanthine and xanthine to generate IMP [41, 42]. After 1 h of treatment, the percentage of cells with IMPDH cytoophidia significantly increased in a dose dependent manner (Fig. 2b, c). Additionally, we also aimed to increase the IMP level by activating the conversion of GMP to IMP, by which intracellular IMP should increase without an elevation in GTP level. To this end we constructed an OFP-P2A-GMPR plasmid, with which the GMPR expression level in transfected cells could be monitored by fluorescence intensity. Normally, IMPDH cytoophidia are detected in < 5% of HeLa cells. Surprisingly, when HeLa cells were transfected with the OFP-P2A-GMPR plasmid, the percentage of cells with cytoophidia dramatically surged to 84 ± 3.7% for cells with high fluorescence intensity. Even the cell population with lower GMPR expression level contained 38 ± 11.5% of cells with cytoophidia (Fig. 2d, f). To exclude possibilities other than the increased GMPR activity to induce cytoophidium formation, we further carried out site-directed mutagenesis to generate plasmids encoding two catalytic dead GMPR mutants, C186A and T188A (PDB 2BWG) [43, 44]. After transfection, the cytoophidia were no longer observed, indicating that filamentation of IMPDH was triggered by increased GMPR activity (Fig. 2e, f). Meanwhile, GMPR overexpression does not affect proliferation rate or IMPDH2 protein levels, implying the intracellular GTP level was not significantly affected by GMPR (Additional file 1: Fig. 2). These findings indicate that IMP accumulation promotes IMPDH cytoophidium formation.

### Knockdown of IMPDH2 in HeLa cells promotes cytoophidium assembly but does not affect proliferation rate

**Fig. 3:**
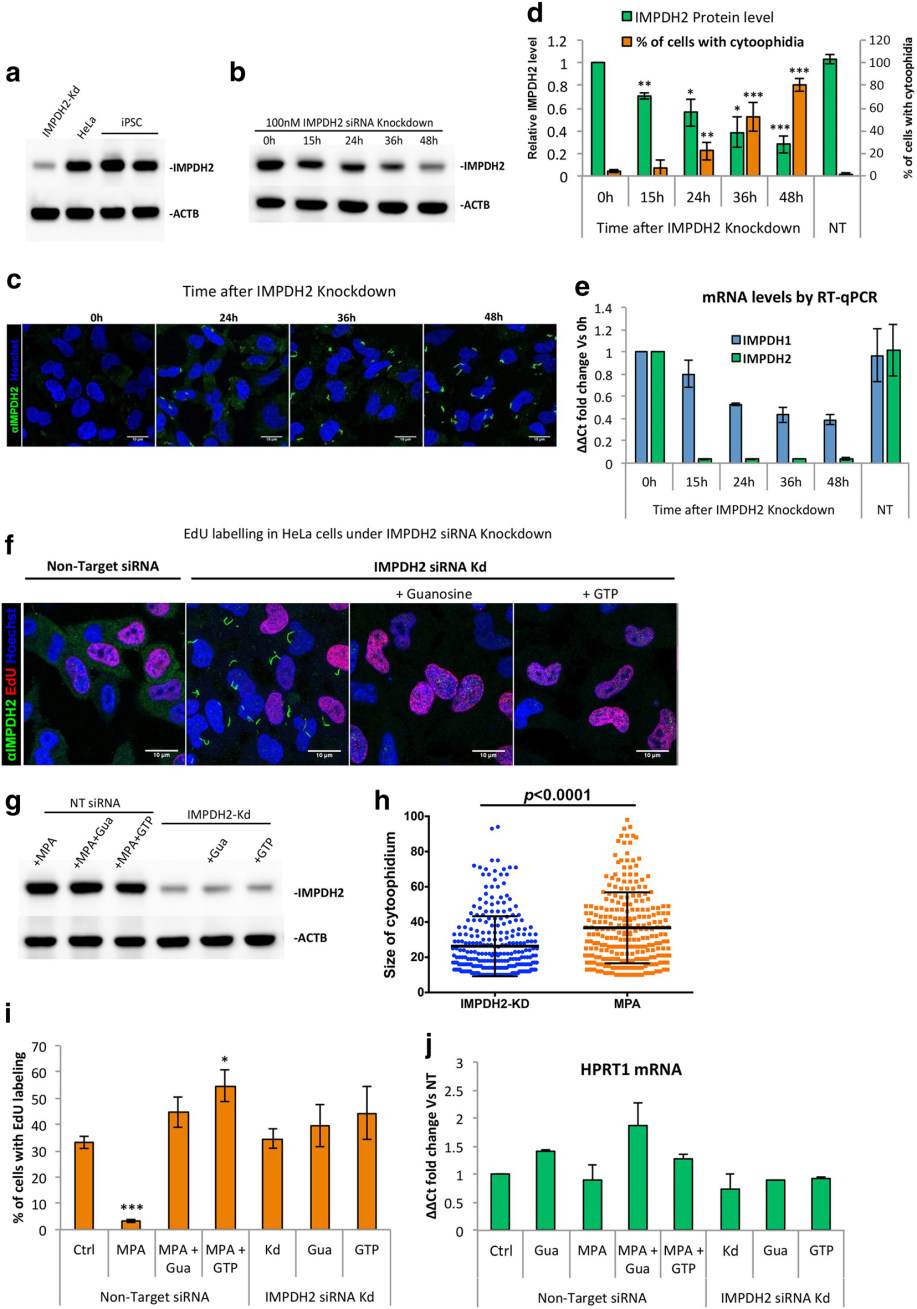
Knockdown of IMPDH2 in HeLa cells promotes cytoophidium assembly but does not affect proliferation rate. **a** IMPDH2 protein levels were analysed by Western blot in total cell extracts of iPSC and HeLa control or under IMPDH2-Kd. **b** IMPDH2 protein levels decrease gradually at various time points after 100 nM IMPDH2 siRNA transfection, reaching 28% in 48 h. **c** Immunofluorescence of HeLa cells after 100 nM IMPDH2 siRNA transfection. **d** Quantitative data of IMPDH2 protein level and percentage of cells with cytoophidia in **b** and **c**. **e** IMPDH1 and IMPDH2 mRNA levels after siRNA transfection. **f** Immunofluorescence of HeLa cells transfected with Non-Target or IMPDH2 siRNA for 48 h. Cells were also treated for 4 h with 1 mM guanosine or 1 mM GTP before fixation. **g** IMPDH2 protein level in cells transfected with Non-Target or IMPDH2 siRNA and treated with 1 mM MPA and/or guanosine/GTP 4 h before lysis. **h** Average size of the cytoophidium in cells under IMPDH2-Kd or MPA treatment. **i** The proportion of EdU-positive cells in **f** and** g**. **j** HPRT1 mRNA level was measured by RT-qPCR in cells transfected with Non-Target or IMPDH2 siRNA and treated with MPA and/or guanosine/GTP. Error bars means SD in **d**, **e** and **h** and SEM in **i** and **j**. For statistics, groups were compared by Student’s *t*-test with zero hour (0 h) in **d** and **e** and with Non-Target in **i** and **j**, *P ≤ 0.05, **P ≤ 0.01, ***P ≤ 0.001

Our results suggest that accumulation of IMP promotes cytoophidium assembly, whereas an increase in GTP disassociates the structure. It has been demonstrated that binding of GTP/GDP on human IMPDH negatively regulates its affinity to IMP [18]. It is reasonable to propose that the presence of the cytoophidium may correlate with an increase of GTP production. We ask if IMPDH would form the cytoophidium to facilitate GTP production when the expression of IMPDH is reduced. That is when the flux of GTP produced by non-polymerized IMPDH is not sufficient to support the cell’s need, filamentation of IMPDH would occur. Since IMPDH2 is the predominant type in most cells, we mainly focused on this isoform. Firstly, we compared the expression level of IMPDH2 in iPSC and HeLa cells, and found a similar expression level (Fig. 3a). However, the metabolic state of iPSCs (~ 67% EdU-positive cells) is assumed to be more active as they have a much higher proliferation rate than HeLa cells, of which 33 ± 5.0% of cells were EdU positive under normal culture conditions (Figs. 1c, 3i, respectively). Notably, as mentioned previously, IMPDH cytoophidia were seen in most of the iPSCs (> 80%) but rarely found in HeLa cells (< 5%) under normal conditions (Figs. 1a, 3d, respectively). We then suppressed IMPDH2 expression in HeLa cells with siRNAs and found that the IMPDH2 protein levels gradually decreased to 56 and 28% in 24 and 48 h, respectively (Fig. 3b and d). The siRNA transfection efficiency was monitored by siGLO green and more than 90% of HeLa cells were successfully transfected (Additional file 1: Fig. 3d). As expected, IMPDH cytoophidia were observed in 22 ± 7.8 and 80 ± 5.7% of cells after 24 and 48 h of IMPDH2 knockdown, respectively (Fig. 3c, d). These cytoophidia were reversible with additional guanosine or GTP, while the levels of IMPDH2 protein were unchanged upon supplementation of additional sources of GTP (Fig. 3f, g). This was also seen on treatment with siRNAs at 1000× lower concentration, although a higher level of IMPDH2 (45% of control) remained in the cells (Additional file 1: Fig. 3a, b).

**Fig. 4:**
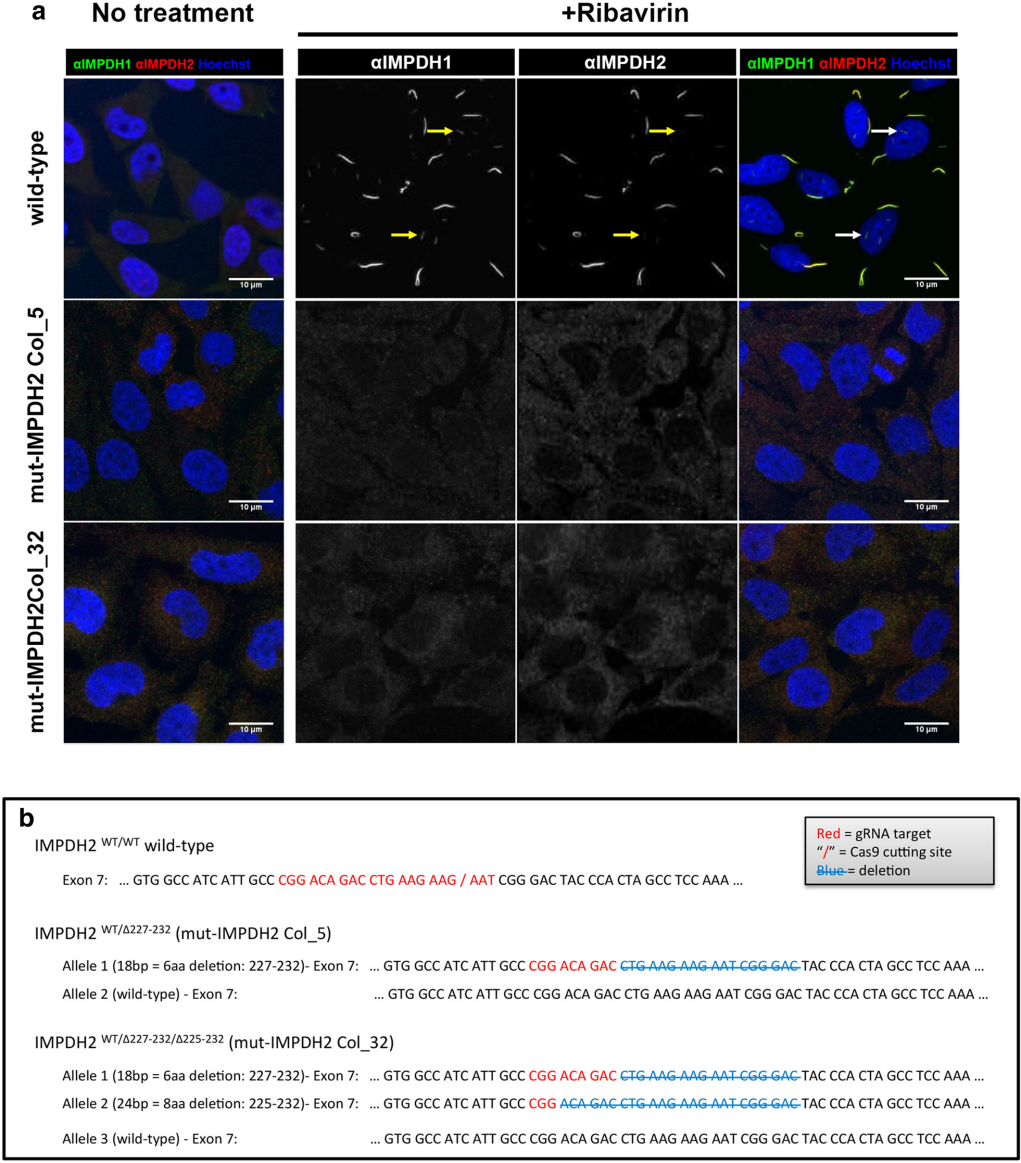
Mutations on CBS domain prevent cytoophidium assembly. **a** Mutant cell lines were treated with 1 mM ribavirin for 4 h and probed with mouse monoclonal anti-IMPDH1 and rabbit polyclonal anti-IMPDH2 antibody to evidence the no-filament phenotype. The arrows in indicate the previously described intranuclear filaments. **b** Cell lines with endogenous mutations induced by CRISPR/Cas9 system on IMPDH2 gene. Sequences showing the genotype of each cell line

We speculated that both IMPDHs isoforms could assemble into the same cytoophidium structure as the labelling of IMPDH1 and IMPDH2 antibodies always colocalised in all IMPDH cytoophidia, including the previously reported intranuclear ones [30] (arrows in Fig. 4a). However, the target of the IMPDH antibodies we used may not be restricted to one specific isoform, since the sequences of two isoforms are highly similar. We then transfected HeLa cells with a combination of plasmids encoding OFP-IMPDH2 and eGFP-IMPDH1, and confirmed that isoforms always colocalise in the cytoophidium structure (Additional file 1: Fig. 3c). Thus, the cytoophidia in IMPDH2 knockdown cells could be composed of remaining IMPDH2 plus the IMPDH1.

**Fig. 5:**
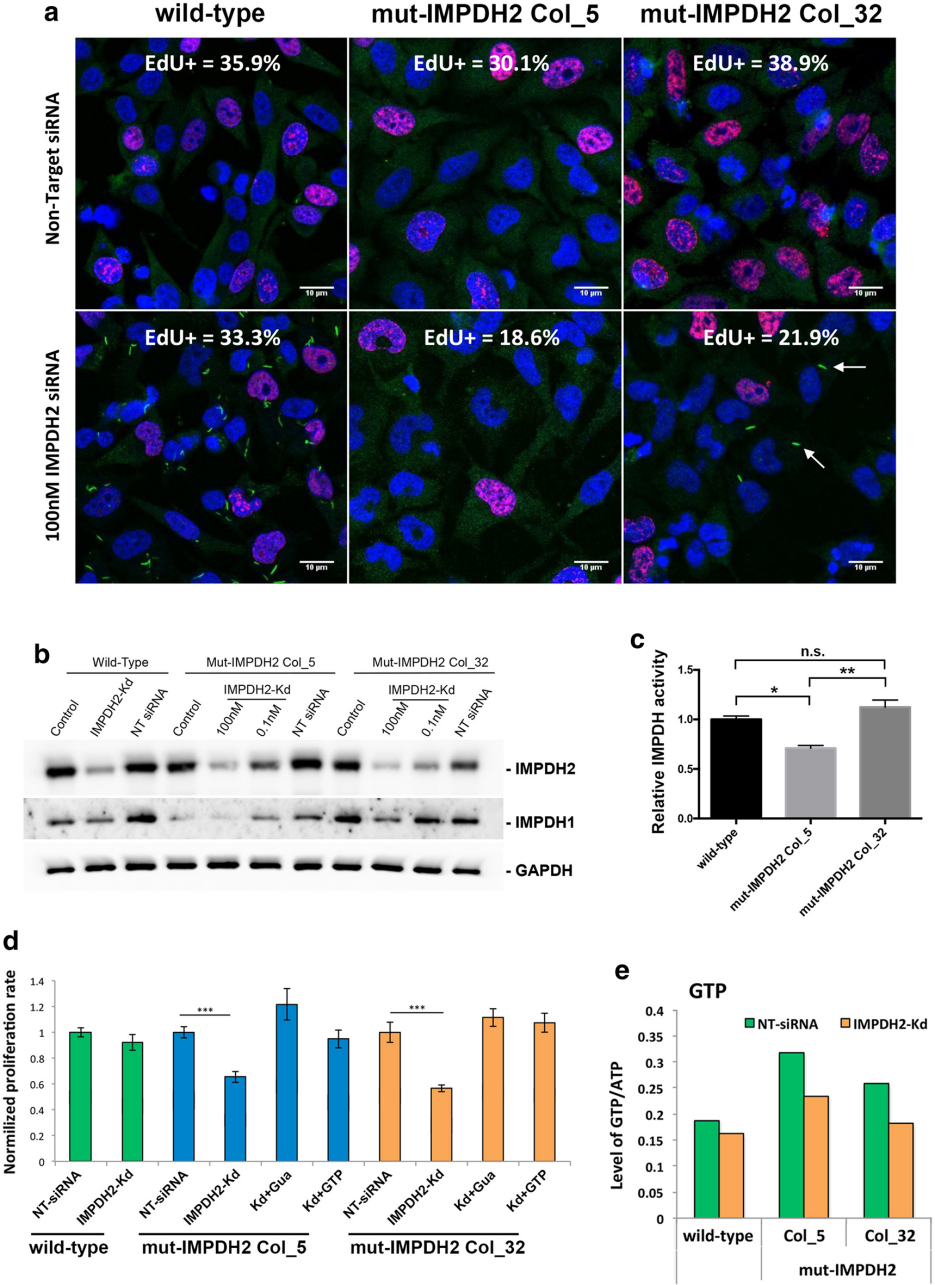
Knockdown of IMPDH2 in cells without cytoophidia affects proliferation rate. **a** Wild-type, mut-IMPDH2 Col_5 and mut-IMPDH2 Col_32 were transfected with Non-Target siRNA (NT) or 100 nM IMPDH2 targeting siRNA for 48 h and labelled with EdU and anti-IMPDH2 antibody. Proportion of EdU-positive cells is presented in the top of each panel. More than 1000 cells were counted and at least three independent experiments were performed for each group. **b** IMPDH1 and 2 protein levels in WT and mut-IMPDH2 Col_5 and Col_32 indicating a decrease in protein levels under increasing doses of siRNA for IMPDH2-Kd. **c** The relative IMPDH activity of cell lysates of wild-type and mutant cell lines. **d** Proliferation rate for each cell line in various conditions was normalized with Non-Target siRNA transfected groups. Cells were transfected with 100 nM IMPDH2 siRNA for IMPDH2-Kd and supplemented with a daily dose of 1 mM guanosine (Gua) or 1 mM GTP. Error bars means SD. Student’s *t*-test, ***P ≤ 0.001. **e** Intracellular level of GTP (standardized with ATP) of wild-type and mutant cells after transfection of Non-Target or IMPDH2 siRNA

Interestingly, the IMPDH2 knockdown cells showed comparable proliferation rate to cells without knockdown, even though the IMPDH2 protein were < 30% of the original level (Fig. 3f, g, i). This suggests that GTP production in IMPDH2 knockdown cells was not severely reduced as seen in MPA-treated cells [45]. To confirm that expression level of IMPDH1 is not increased to compensate for the loss of IMPDH2, we measured the mRNA levels of IMPDH1 and found a significant decrease after 48 h of transfection with IMPDH2 siRNAs (Fig. 3e). This could be caused by the IMPDH2 siRNAs since one of the four siRNA fragments applied in this experiment has a 14 bp match with IMPDH1 mRNA. However, clearly IMPDH1 expression does not increase to compensate the decrease of IMPDH2, also evidenced by the IMPDH1 protein levels (Fig. 5b). On the other hand, guanine nucleotides can be produced by salvage pathway as well. To evaluate whether GTP production could be compensated for by an upregulated salvage pathway, we measured the mRNA level of HPRT1 and found no difference when cells were transfected with IMPDH2 siRNAs alone or together with guanosine or GTP supplementation (Fig. 3i). As control, cells were treated with IMPDH-inhibitor MPA for 2 days and supplied daily with 1 mM of HPRT substrate guanosine. HPRT1 mRNA increased 1.85 times compared with the control (Fig. 3i). These results show that in an IMPDH2 knockdown situation, the remaining amount of IMPDH proteins (< 30% of normal) would tend to form the cytoophidium and GTP production is still adequate to support normal cell proliferation.

### Mutation in CBS domain of IMPDH2 impairs cytoophidium assembly

It has been suggested that the CBS domain modulates IMPDH polymerisation. Multiple point mutations on human IMPDH1 have been identified associated with the retinopathy adRP10. Among them, R224P and D226 N mutations can alter IMPDH1 filament-forming properties in human cells [20]. In addition, purified human IMPDH1 D226 N protein tends to comprise large aggregates, which are formed by numerous intertwined IMPDH1 fibres in vitro [17]. However, none of these adRP10-related point mutations has been observed in IMPDH2. Perhaps because IMPDH2 is the predominant isoform in most tissues, therefore any abnormality may lead to severe defects, even embryonic lethality [46, 47]. We wondered whether such mutations on IMPDH2 would result in the same consequences as on IMPDH1 regarding filament-forming properties.

We firstly generated R224P, D226 N and R231P myc-IMPDH1 mutants. All of them formed irreversible cytoophidia in transfected HEK 293T cells when treated with guanosine (Additional file 1: Fig. 4a, c). Next, we examined the same mutations on myc-IMPDH2. Similar to the IMPDH1 mutants, D226 N and R231P myc-IMPDH2 mutants formed irreversible cytoophidia that could not be disassembled by the given guanosine treatment. Surprisingly, however, the myc-IMPDH2 R224P mutant abolished the cytoophidia in transfected cells under the condition of supplementation with MPA (Additional file 1: Fig. 4b, c). These results indicate the distinct features of the IMPDH1 and IMPDH2 CBS domains, and also suggest that it is possible to prevent IMPDH cytoophidium formation by introducing mutations in the IMPDH2 CBS domain.

### Endogenous mutations on CBS domain prevent cytoophidium assembly

In order to determine if forming the cytoophidium is necessary for facilitating GTP production in certain conditions, we sought to establish a cell model with no capability to form IMPDH cytoophidia. To achieve this, we applied CRISPR/Cas9 genome editing technique to induce mutagenesis in the CBS domain of endogenous IMPDH2 of HeLa cells with a sgRNA targeting exon 7, which corresponds to amino acid 224–230. Out of over 150 single-cell clones, two colonies exhibited the ‘no-cytoophidium’ phenotype under induction with ribavirin, MPA and DON (Fig. 4a). With sequencing analysis, we confirmed that one colony (mut-IMPDH2 Col_5) had a deletion of six residues from 227 to 232 in one allele and no mutation in the other (Fig. 4b). The other colony (mut-IMPDH2 Col_32) had a deletion of eight residues (Δ225–232) in one allele, a deletion of six residues (Δ227–232) in another allele, and at least one extra allele of wild-type IMPDH2 (Fig. 4b). Under normal culture conditions, no defects in cell proliferation rate and IMPDH2 levels have been observed in both colonies (Fig. 5a, b).

Although it has been reported previously that truncation of the entire CBS domains has no impact on IMPDH activity in vitro, point mutations is still possible to change certain properties of the enzyme, including catalytic activity [18, 19]. To determine whether the deletions in CBS domain could affect IMPDH2 activity, we conducted an in vitro IMPDH activity analysis for cell lysates of wild type, mut-IMPDH2 Col_5 and mut-IMPDH2 Col_32 cells. While mut-IMPDH2 Col_5 showed 30% lower activity, the IMPDH activity of mut-IMPDH2 Col_32 was similar to that of the wild-type cells, within error (Fig. 5c). All cell lines showed indistinguishable IMPDH2 level, while mut-IMPDH2 Col_5 cells had a lower amount of IMPDH1 and mut-IMPDH2 Col_32 cells expressed more IMPDH1 than wild-type cells (Fig. 5b). Such differences in in vitro catalytic activity assay may be attributed to the variances of their IMPDH1 level. Since mut-IMPDH2 Col_32 cells, which have similar expression level of IMPDHs as wild-type cells, showed comparable activity with wild-type cells, we suggest that the deletions in CBS domain do not affect IMPDH2 activity.

To ensure that the ‘no-cytoophidium phenotype’ in mut-IMPDH2 Col_5 and mut-IMPDH2 Col_32 cells is truly the result of the deletion of six residues (Δ227–232) in part of IMPDH2 proteins, we also overexpressed the mutant IMPDH2 in wild type HeLa cells and observed the absence of cytoophidium assembly under ribavirin treatment (Additional file 1: Fig. 5).

### Cytoophidium assembly is important for keeping normal proliferation rate of HeLa cells when IMPDH2 expression is suppressed

Our results show that under IMPDH2 knockdown, cytoophidium assembles and the proliferation rated of the cells was not affected. We ask if the same would happen with the ‘no-cytoophidium phenotype’ cell lines. Thus, transfection of IMPDH2 siRNAs in mutant HeLa cell lines was carried and nucleotide levels and their proliferation rates were analysed. Interestingly, 48 h after IMPDH2 knockdown, with the lack of cytoophidia, the percentage of EdU-positive cells in mut-IMPDH2 Col_5 and mut-IMPDH2 Col_32 declined to 66 ± 4.4 and 57 ± 2.7% of the original proportion (normalized to 100% for each cell line), whereas the proliferation rate of wild-type cells was not affected under the same condition (Fig. 5a, d). Notably, we observed small cytoophidia in 9.7 ± 1.7% of IMPDH2 siRNA-transfected mut-IMPDH2 Col_32 cells (arrows in Fig. 5a). We speculated that these cytoophidia observed under the IMPDH2 knockdown condition could be formed mainly by IMPDH1 and wild-type IMPDH2 when the amount of mutant IMPDH2 protein, which interferes with filamentation, was no longer enough for preventing cytoophidium assembly (Fig. 5a).

To test whether the decrease in proliferation rate, caused by IMPDH2 knockdown, in mut-IMPDH2 Col_5 and mut-IMPDH2 Col_32 cells is a consequence of an inadequate GTP pool, we analysed nucleotide levels in given conditions with UPLC. When IMPDH2 level decreased, the GTP/ATP ratio of wild-type HeLa cells slightly declined from 0.187 to 0.161 (ΔGTP/ATP = 0.026). Moreover, the GTP/ATP ratio of mut-IMPDH2 Col_5 and mut-IMPDH2 Col_32 cells reduced 0.843 and 0.760, respectively, which are 3.22 and 2.9-fold of ΔGTP/ATP of wild-type cells (Fig. 5e and Additional file 1: Fig. 6a). Meanwhile, the CTP/ATP and UTP/ATP levels of all three cells lines were only slightly affected (Additional file 1: Fig. 6b). Next, we supplemented guanosine or GTP in the medium following IMPDH2 knockdown in order to compensate the defect of GTP production. With treatments with guanosine or GTP, proliferation rates of two mutant colonies restored to a normal level (Fig. 5d).

**Fig. 6:**
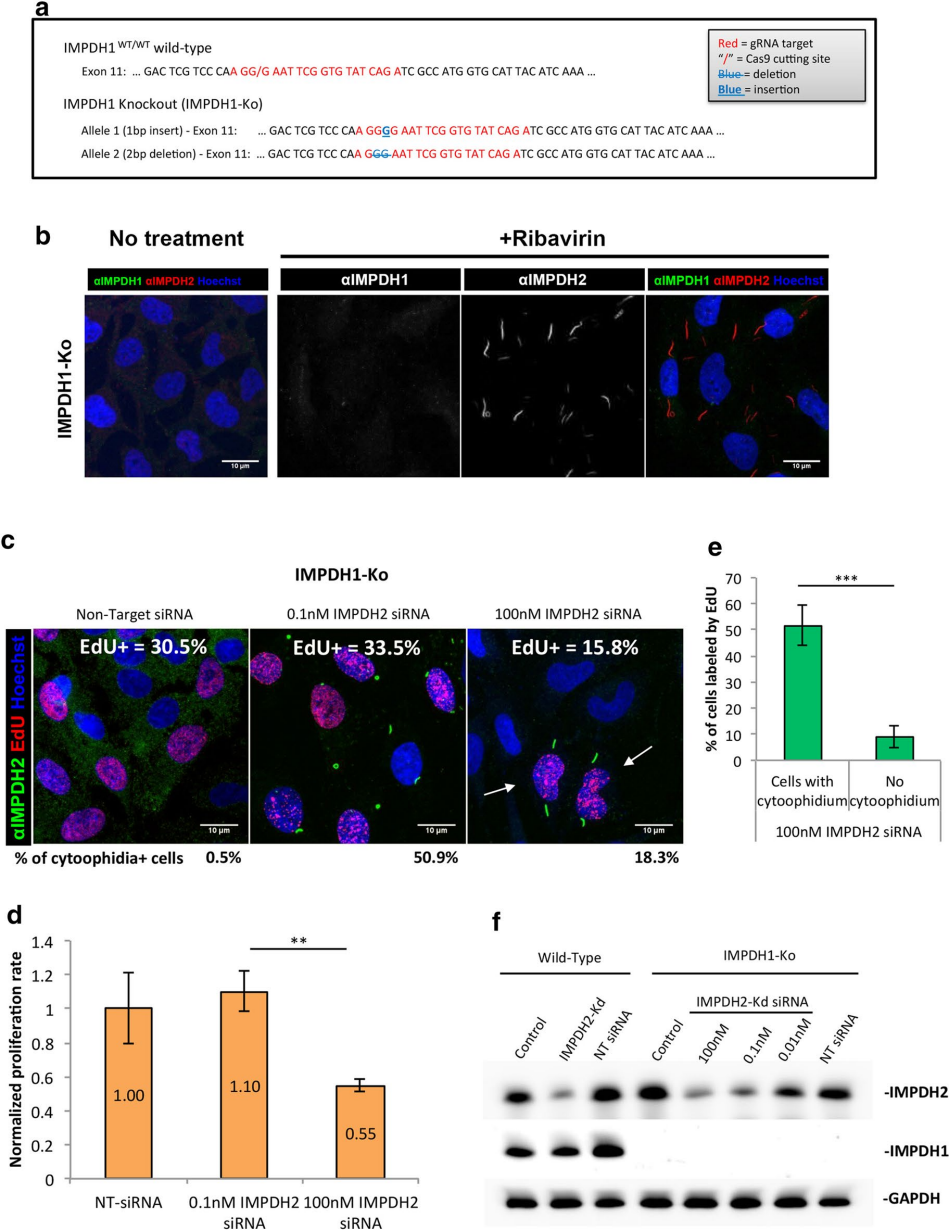
Knockdown of IMPDH2 in IMPDH1-KO cells impairs cell proliferation. **a** Sequences showing the Cas9 cutting site and indels in IMPDH1 gene of IMPDH1-KO cells. **b** Immunofluorescence of IMPDH1-KO cell line after treatment with 1 mM ribavirin for 4 h. **c** IMPDH1-KO cells were transfected with NT siRNA or 0.1 or 100 nM of IMPDH2 siRNA, and labelled with EdU and anti-IMPDH2 antibody. Proportion of cells labelled by EdU is presented in the top of each panel and proportion of cells containing cytoophidia is presented below each panel. More than 700 cells were counted for each group. **d** Proliferation rate of each IMPDH1-KO group normalized with the group transfected with Non-Target siRNA. **e** Quantitative data of cells labelled by EdU in **c**. **f** IMPDH2 protein levels in WT and IMPDH1-KO cells indicating a decrease in protein levels with various doses of siRNA for IMPDH2 knockdown. No IMPDH1 was found in IMPDH1-KO cell line. For statistics, groups were compared by Student’s *t*-test as indicated, **P ≤ 0.01, ***P ≤ 0.001

We have demonstrated that IMPDH1 and IMPDH2 would form the same filament structure (Additional file 1: Fig. 3c), suggesting that both isoforms could be regulated by the cytoophidium. We wondered whether IMPDH1 is mainly supporting sufficient GTP production when the IMPDH2 level is massively reduced. Thus, we generated an IMPDH1 knockout cell line (IMPDH1-Ko) by targeting exon 11 (Fig. 6a). The proliferation rate of this cell line was similar to that of wild-type cells (Figs. 5a, 6c). Furthermore, in IMPDH1-Ko cells cytoophidia were only observed under induction, suggesting a dispensable role of IMPDH1 in normal cell metabolism (Fig. 6b).

We then transfected IMPDH1-Ko cells with IMPDH2 siRNAs at low (0.1 nM) and high (100 nM) concentration and analysed the proliferation rate. When cells were transfected with 0.1 nM siRNAs, cytoophidia were observed in 50.9 ± 21.7% of cells and no defect in cell proliferation was seen (Fig. 6c, d). However, when cells were transfected with the high dose of siRNAs, only 18.3 ± 4.9% of cells exhibited cytoophidia and cell proliferation significantly decreased to 55 ± 3.9% of the original level (Fig. 6c, d). Notably, since there was no IMPDH1 present in the cells, these cytoophidia were composed of remaining IMPDH2 (Fig. 6f). Moreover, a clear correlation between cells with cytoophidia and proliferating cells (EdU positive) was found (arrows in Fig. 6c). While 51 ± 7.8% of cytoophidium expressing cells were labelled by EdU, only 9 ± 4.2% of cells without cytoophidia were EdU positive (Fig. 6e). Taken together, our results suggest that the cytoophidium structure is of the utmost importance for upregulating IMPDH activity and maintaining the intracellular GTP level during certain circumstances, such as decreased IMPDH protein level or an increase of GTP consumption, thereby coordinates the supply and demand of guanine nucleotides.

## Discussion

IMPDH plays a key role in purine nucleotide biosynthesis and hence contributes to controlling cell metabolism and proliferation. Therefore, IMPDH has drawn attention as a promising target in various clinical applications. For example, IMPDH inhibitors, such as MPA and ribavirin, are widely used as an immunosuppressant and for treatment of HCV infection, respectively. These drugs, and other conditions that disturb de novo GTP production, are known to induce massive cytoophidia formation in vitro or in vivo [40]. As a result, the IMPDH cytoophidium has been considered as a consequence of IMPDH inhibition or GTP insufficiency [15, 20, 24, 48]. However, our previous report suggests that the assembly of cytoophidia reflects upregulation of GTP synthesis [31]. In the current study, we provide multiple lines of evidence that IMP promotes IMPDH filamentation. Since it has been shown that the IMP binding affinity of IMPDH could be greatly reduced by the conformational change due to GDP/GTP binding at the CBS domain [18], we propose that the formation of the IMPDH cytoophidium is positively correlated with the ratio of intracellular IMP to guanine nucleotides.

In mouse iPSCs, active glycolysis and de novo nucleotide biosynthesis are essential features for supporting their rapid proliferation, although it is not necessarily specific to a pluripotent state. The balance between the high levels of GTP production and consumption is suggested as the reason for the presence of IMPDH cytoophidia in ~ 80% of iPSCs. Any treatment that interrupts this balance could perturb IMPDH aggregation. For instance, cell cycle arrest and GTP supplementation, which may result in GTP accumulation, significantly decreased the proportion of cells with cytoophidia. In contrast, both treatments with allopurinol or overexpression of GMPR may facilitate the generation of IMP through salvage and recycling pathways and thereby induce cytoophidium formation in HEK 293T cells and HeLa cells.

On the other hand, it has been demonstrated that the CBS domain of IMPDH is dispensable for enzyme activity in vitro, but participates in the regulation of nucleotide pools and also IMPDH filamentation [18–20]. Several IMPDH1 mutations in CBS domains are known to be related to retinopathy adRP10 [49]. Among them, the R224P and D226 N mutants were shown to perturb IMPDH clustering in cells [20]. Herein, we found that another adRP10-related IMPDH1 mutant, R231P, also enforces IMPDH to assemble irreversible cytoophidia. Interestingly, despite these mutant residues being conserved in IMPDH2, we observed a different effect from IMPDH2 R224P. Instead of assembling into the cytoophidium, IMPDH2 R224P prevented clustering of both isoforms. These findings support the notion that the CBS domain plays a key role in mediating cytoophidium assembly. A natural product sanglifehrin A (SFA), has been shown to form a complex with protein cyclophilin A (PPIA) inside human cells [50]. The PPIA-SFA complex is able to bind with IMPDH2, but not IMPDH1, at the CBS domain and thereby suppress proliferation of lymphocytes. Although the mechanism results in cell growth inhibition and whether the binding of PPIA-SFA would disturb IMPDH filamentation is currently unclear, this suggests that it is possible to modulate IMPDH filamentation with molecules targeting the CBS domain.

The octameric state of IMPDH has been suggested to play an important role in allosteric regulation and formation of IMPDH fibres [17, 18, 51]. Structures of two types of octamers of human IMPDH1 have been revealed and it is suggested that these two octameric architectures could pile up to form protein fibres [17]. These IMPDH fibres could further associate together forming a higher order structure, which is very likely the cytoophidium in cells [17, 30].

Recently, a publication by Anthony et al. [35] shows that the IMPDH cytoophidium accommodates both catalytically active and inactive conformations of the enzyme. They also identified three additional point mutations in human IMPDH2 that interfere with cytoophidium assembly, while S275L overexpression results in permanent filament formation, Y12A and R356A disrupt filamentation, even in the presence of MPA. No difference in catalytic activity was detected in all three mutant IMPDH2 proteins in vitro when compared with wild-type IMPDH2. Similarly, HEK293 cells overexpressing S275L mutant and S275L/Y12A double mutant IMPDH2 proteins exhibited indistinguishable guanine nucleotide biosynthetic rate. However, overexpression of IMPDH may not be the best way to study such effects on catalytic activity as the GTP level might be always above the normal level, which could bring negative feedback to the biosynthetic pathway and suppress IMPDH activity [51].

Herein, we generated mutant HeLa cell lines having a deletion in the CBS domain of endogenous IMPDH2 and characterised these mutant cells as being unable to form the cytoophidium. Although no growth defect was found under normal conditions, these mutant cells could not sustain the normal GTP pool and cell proliferation rate when the expression of IMPDH2 was suppressed. Despite the transcriptional function of IMPDH has been demonstrated [52], our data suggest that assembly of the IMPDH cytoophidium is correlated with its catalytic function. Since a growth defect in mutant cells was only seen when IMPDH2 was knocked down, the IMPDH cytoophidium seems not to be essential for IMPDH being active but important for another aspect of enzyme regulation. Polymerization of active acetyl-CoA carboxylase has been described more than 30 years ago [53]. More recently, the substrates promoted polymerization of human CTPS1, which is not essential for CTPS1 being active, has been revealed as to increase its catalytic activity [33]. It is suggested that an active conformation of human CTPS1, which may facilitate ammonia transfer between two active sites, is stabilized in the filaments. This may keep CTPS in a primed state of maximal activity under certain conditions in which higher CTP production is required for the cell [33]. We propose that formation of the IMPDH cytoophidium have a similar purpose to facilitate the catalytic reaction of IMPDH by putting the enzyme into a hyperactive state in response to an elevated ratio of IMP to guanine nucleotides. Indeed, the model of IMPDH polymer demonstrated by prior study implies the polymer may coordinate IMPDH octamer conformational changes, although further analysis is still needed to reveal how that affects the catalytic activity of IMPDH [35].

## Conclusion

In summary, we show that the formation of the IMPDH cytoophidium is controlled by the levels of IMP and GTP, by which the production of and demand for guanine nucleotides can be exquisitely coordinated. Moreover, cytoophidium assembly in mouse iPSCs correlates with the high proliferation rate observed in those cells. Cytoophidium assembly may be involved in upregulation of the whole system of de novo purine nucleotide biosynthesis, which these cells heavily depend on. In addition, the assembly of the cytoophidium may induce a hyperactive state in both IMPDH1 and IMPDH2 under specific cell metabolic conditions, as for example when the amount of IMPDH2 was artificially decreased. Taken together, our findings shed light on the fundamental regulation and function of the IMPDH cytoophidium in mammalian cells, providing new insight into the control of IMPDH activity and guanine nucleotide homeostasis. Considering the important role of IMPDH in clinical applications, our results propose a new perspective for drug development in the future. Further studies are needed to elucidate how filamentation changes the IMPDH protein folding structure and how such alterations affect enzyme activity by, for example, facilitating the binding of IMPDH to its substrate IMP.

## Additional file

**Additional file 1.** An additional file containing six Figures and one Table is provided, together with the respective figure legends. The Additional file is composed of: **Figure S1**. dCTP treatment does not affect IMPDH-based cytoophidia in iPSCs; **Figure S2**. Proliferation rate not affected by GMPR overexpression; **Figure S3**. IMPDH2 knockdown induces cytoophidium assembly; **Figure S4**. Mutations on CBS domain of IMPDH isoforms result in distinct effects on cytoophidium formation; **Figure S5**. Deletion of the 6 residues from 227 to 232 in myc-IMPDH2 prevents cytoophidium assembly; **Figure S6**. Intracellular GTP level (but not UTP or CTP) significantly drops in no-cytoophidium mutant cells after IMPDH2 knockdown. **Table S1**. Nucleic acid oligos used in the experiments, including primers, siRNAs and sgRNAs for CRISPR/Cas9 genome editing.
