# Supplementary material for: IMP/GTP balance modulates cytoophidium assembly and IMPDH activity
Source: Cell Div. 2018 Jun 15;13:5. doi: 10.1186/s13008-018-0038-0 (PMC6004095; doi:10.1186/s13008-018-0038-0)
Supplement: Supplementary file 1 — Additional file 1. An additional file containing six Figures and one Table is provided, together with the respective figure legends. The Additional file is composed of: Figure S1. dCTP treatment does not affect IMPDH-based cytoophidia in iPSCs; Figure S2. Proliferation rate not affected by GMPR overexpression; Figure S3. IMPDH2 knockdown induces cytoophidium assembly; Figure S4. Mutations on CBS domain of IMPDH isoforms result in distinct effects on cytoophidium formation; Figure S5. Deletion of the 6 residues from 227 to 232 in myc-IMPDH2 prevents cytoophidium assembly; Figure S6. Intracellular GTP level (but not UTP or CTP) significantly drops in no-cytoophidium mutant cells after IMPDH2 knockdown. Table S1. Nucleic acid oligos used in the experiments, including primers, siRNAs and sgRNAs for CRISPR/Cas9 genome editing. [file 13008_2018_38_MOESM1_ESM.pdf]

Keppeke and Chang et al., ADDITIONAL FILE 1

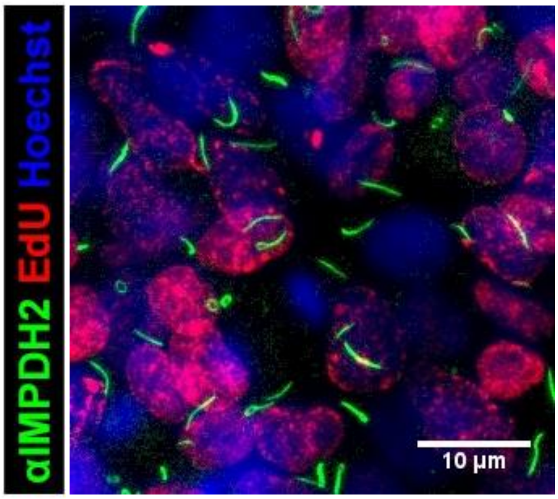

Figure S1. dCTP treatment does not affect IMPDH-based cytoophidia in iPSCs. Cells were treated with 1 mM dCTP for 4 hours and labelled with anti-IMPDH2 antibody and EdU.

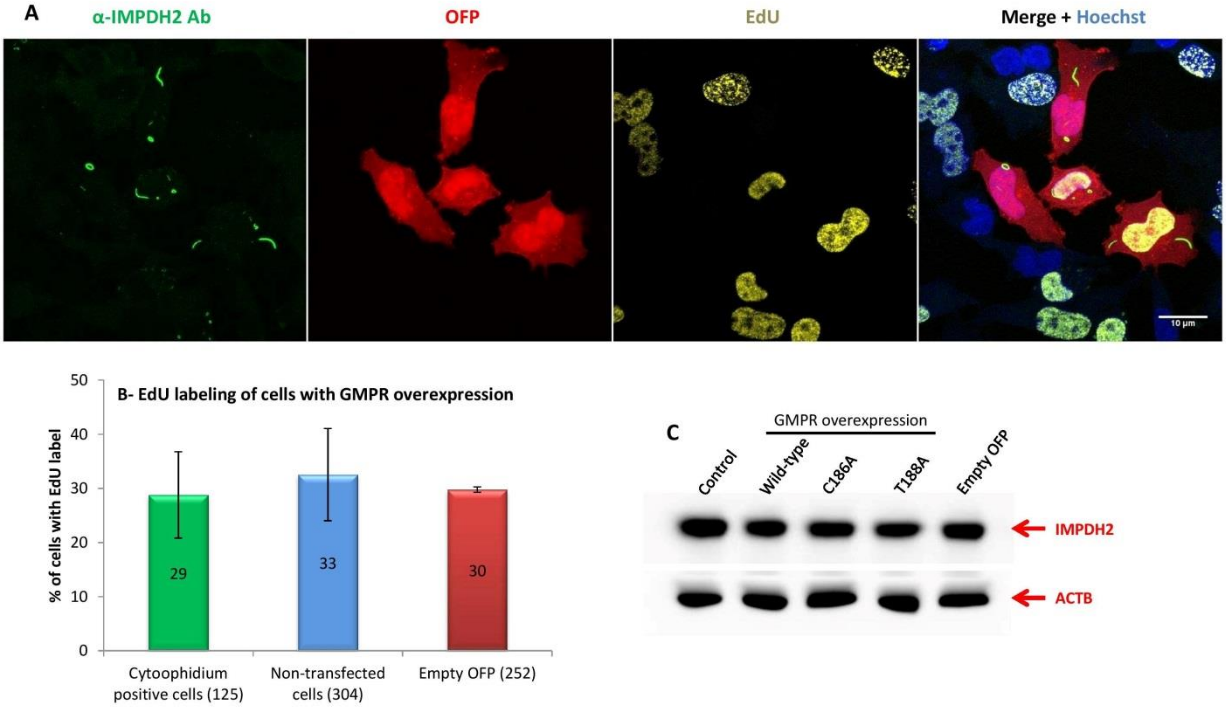

Figure S2. Proliferation rate not affected by GMPR overexpression. (A) Hela cells expressing OFP-P2A-GMPR for 48 hours were labelled by EdU and anti-IMPDH2 antibody. (B) Mean ± SD of proportion of EdU labelling in cells presenting cytoophidia induced by GMPR overexpression, cells without transfection from the same experiment, or cells independently transfected with an empty OFP plasmid as control. Amount of cells counted in presented in brackets. (C) IMPDH2 protein level in cells transfected with different GMPR plasmids or empty OFP plasmid for 48 hours.

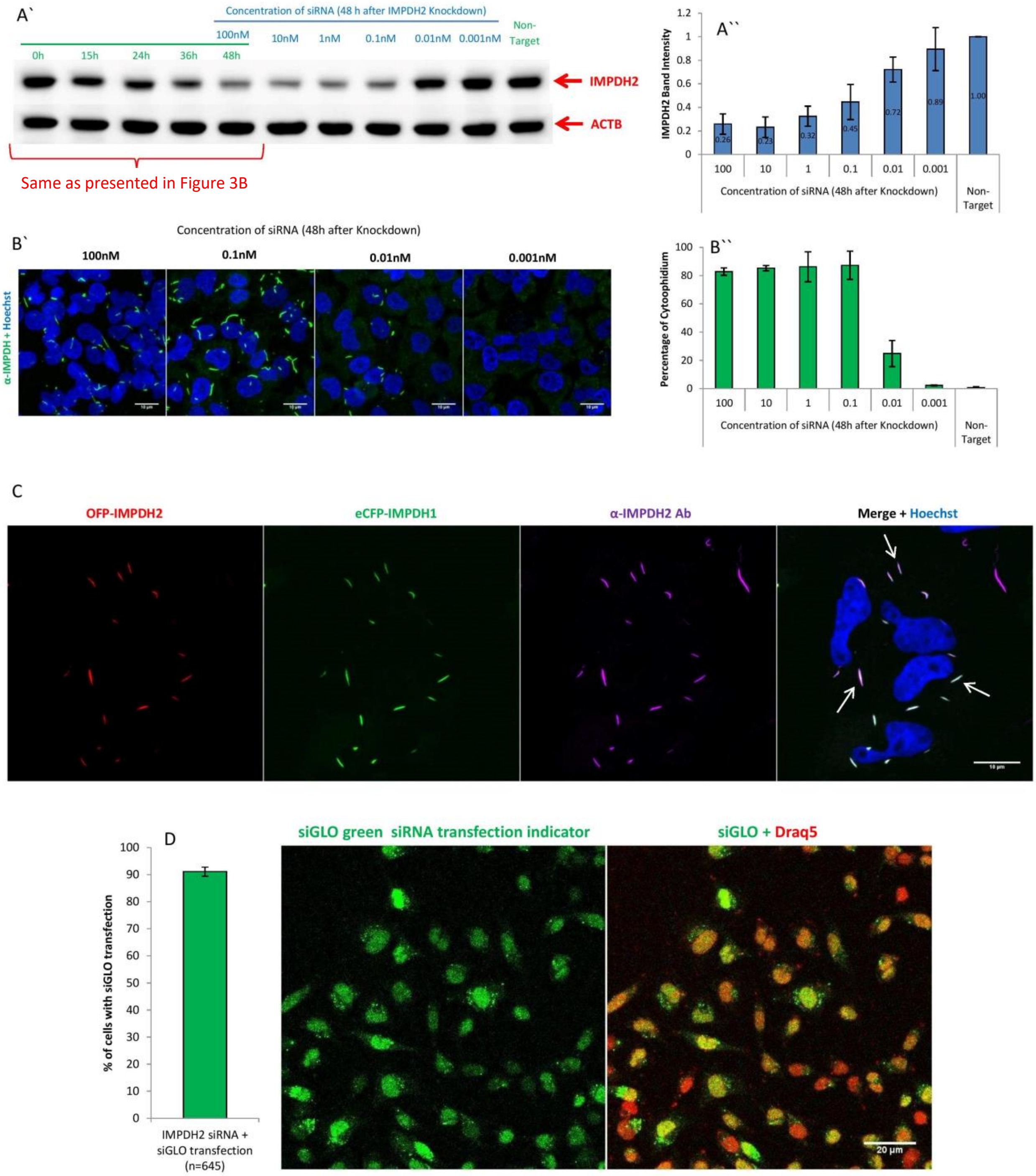

Figure S3. IMPDH2 knockdown induces cytoophidium assembly. (A) IMPDH2 protein levels in cells submitted to IMPDH2-Kd with different gradually decreasing concentrations of siRNA. (A’) Quantitative data of (A). (B) Proportion of cytoophidium in cells submitted to IMPDH2-Kd with different gradually decreasing concentrations of siRNA. (B’) Quantitative data of (B). (C) HeLa cells transfected with OFP-IMPDH2 and eCFP-IMPDH1 for 24 hours. Cells were treated with 500 µM of ribavirin for 3 hours before fixation and probed with anti-IMPDH2 antibody. Arrows indicate cytoophidium labelled by OFP-IMPDH2, eCFP-IMPDH1 and anti-IMPDH2 antibody. (D) siGLO green was used to track siRNA transfection efficiency, which was >90%.

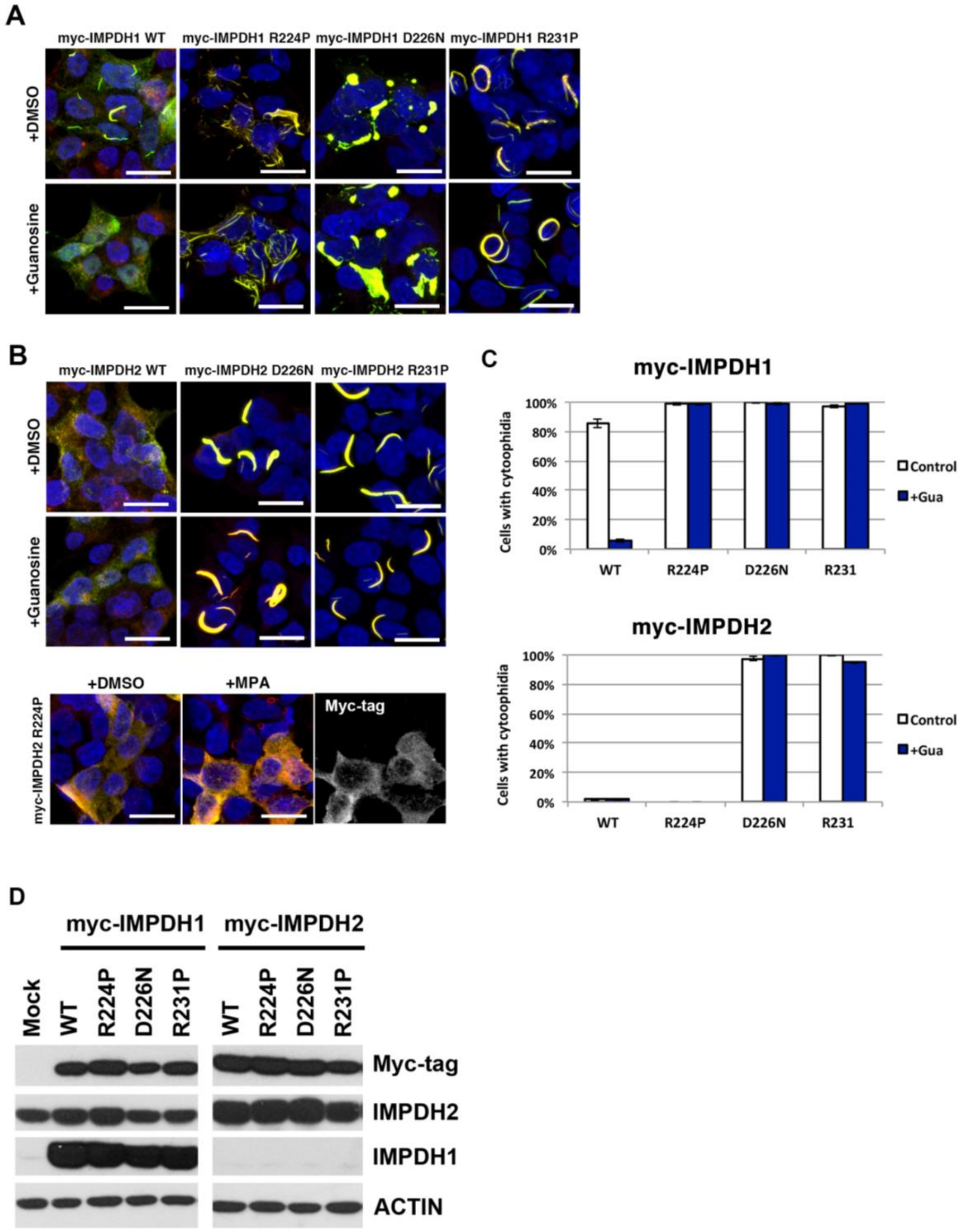

Figure S4. Mutations on CBS domain of IMPDH isoforms result in distinct effects on cytoophidium formation. (A) Immunofluorescence for myc-tag antibody (green), IMPDH2 antibody (red) and DAPI (blue) in HEK 293T cells transfected with wt or mutant myc-IMPDH1 plasmids. Cells were treated with DMSO or guanosine (100 μM) for 1 hour before fixation (Scale bars = 20 μm). (B) Immunofluorescence for myc-tag antibody (green), IMPDH2 antibody (red) and DAPI (blue) in HEK 293T cells transfected with wt or mutant myc-IMPDH2 plasmids. Cells were treated with DMSO, guanosine (100 μM) or MPA (100 μM) for 1 hour before fixation (Scale bars = 20 μm). (C) Mean ± SEM quantitative results of cells with IMPDH cytoophidia for groups shown in (A) and (B). (D) Expression levels for myc-IMPDH1 and myc-IMPDH2 wt and mutants.

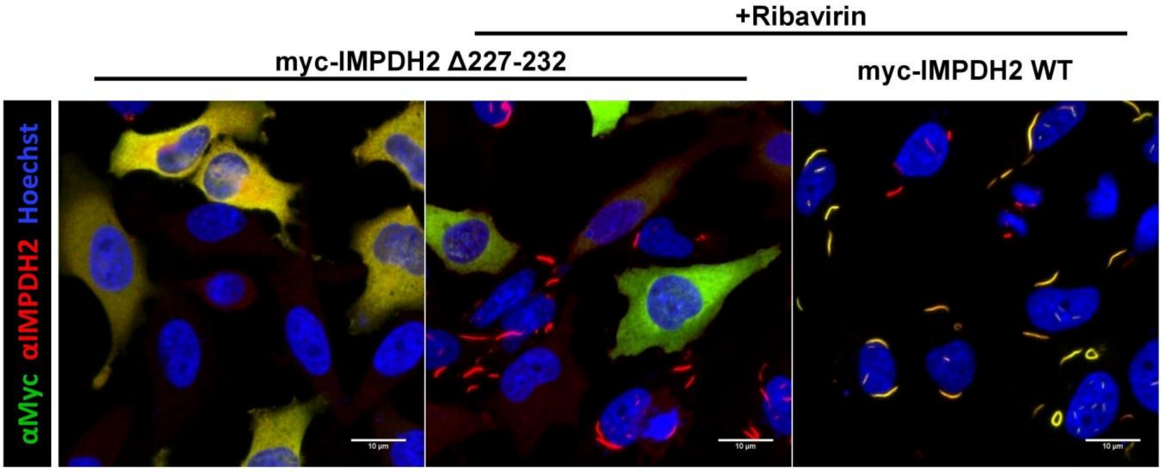

Figure S5. Deletion of the 6 residues from 227 to 232 in myc-IMPDH2 prevents cytoophidium assembly. The myc-IMPDH2 Δ227-232 plasmid was delivered into HeLa cells for 24 hours followed by 1 mM ribavirin treatment for 4 hours before fixation. Cells were labelled with myc-tag antibody (green), IMPDH2 antibody (red) and Hoechst (blue). While cytoophidia were observed in cells with myc-IMPDH2_WT overexpression under ribavirin treatment, no cytoophidium was observed in cells expressing myc-IMPDH2 Δ227-232 mutant under the same condition.

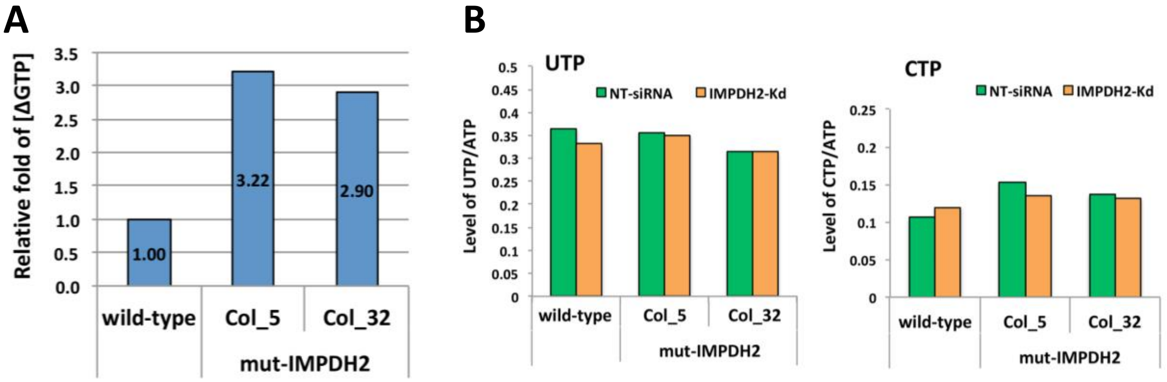

Figure S6. Intracellular GTP level (but not UTP or CTP) significantly drops in no-cytoophidium mutant cells after IMPDH2 knockdown. (A) The difference of GTP level (ΔGTP) between Non-Target and IMPDH2 siRNA transfected conditions in wild-type and mutant HeLa cell lines. (B) Intracellular level of UTP and CTP (standardized with ATP) of wild-type and mutant cells after transfection of Non-Target or IMPDH2 siRNA.

Table S1.

Primers used for RT-qPCR (5’ -> 3’)			
	Target gene	Forward Primer	Reverse Primer
Primers for human HeLa cDNA	IMPDH1	TTCGTGCCCTACCTCATAGC	ATGGACCGAAGGACAGACAG
	IMPDH2	AGTGGCTCCATCTGCATTACG	ACCTTGTACACTGCTGTTGCTTG
	GAPDH	AACGGGAAGCTTGTCATCAATGGAAA	GCATCAGCAGAGGGGGCAGAG
	HPRT1	AGGCGAACCTCTCGGCTTTC	CTAATCACGACGCCAGGGCT

sgRNAs for CRISPR/Cas9 genome editing (5’ -> 3’)	
Target IMPDH1 exon 11	TCTGATACACCGAATTCCCT
Target IMPDH2 exon 7	CGGACAGACCTGAAGAAGAAT

Primers for PCR amplification of the DNA regions targeted by CRISPR/Cas9 (5’ -> 3’)		
	Forward Primer	Reverse Primer
IMPDH1	AGACGTGGAGGAGAACCCTGGACCT<br>TGAAGGACAGAAAAGGGTTACTCAT	CCCGAATTCGGCGGCCGCTCTAGAT<br>ACAGTTTTCATAGGATTTGAGGGGT
IMPDH2	AGACGTGGAGGAGAACCCTGGACCT<br>AACTGGTCATAGTCGATGACTGGCC	CCCGAATTCGGCGGCCGCTCTAGAT<br>GTAAGTCTCAGACTGTGATGTGGCAGC

cDNA cloning for overexpression (5’ -> 3’)		
	Forward Primer	Reverse Primer
Myc-IMPDH1	TGACGCGTATGGAACAAAAACTCATCTCAG<br>AAGAGGATCTGATGGCGGACTACCTGATCA	CGCGGCCGCTGTCCTCAG<br>TACAGCCGCT
Myc-IMPDH2	TGACGCGTATGGAACAAAAACTCATCTCAG<br>AAGAGGATCTGATGGCCGACTACCTGATTA	CGCGGCCGGGTGTGCTGGATCCCTTTTC
GMPR	AGACGTGGAGGAGAACCCTGGACCT<br>ATGCCCCGCATAGATGCGGACCTCAA	CCCGAATTCGGCGGCCGCTCTAGAT<br>GCTGCTTTGTCCCCAGGGTTAGCTG

Predesigned siGENOME SMARTpool siRNA for IMPDH2 knockdown (5’ -> 3’)	
GGACAGACCUGAAGAAGAA	GCACGGCGCUUUGGUGUUC
GGAAAGUUGCCCAUUGUAA	CUAAAGAAAUAUCGCGGUA
